# Top-Down Design Approach of Lightweight Composite Battery Pack Enclosure for Electric Vehicles Based on Numerical Modeling and Topology Optimization

**DOI:** 10.3390/polym17212897

**Published:** 2025-10-29

**Authors:** Xin Zhang, Qiang Lin, Ying Xiao, Liyong Jia, Tiantian Yang, Lei Wang, Quanjin Ma, Bing Wang

**Affiliations:** 1 Fujian Provincial Key Laboratory of Terahertz Functional Devices and Intelligent Sensing, School of Mechanical Engineering and Automation, Fuzhou University, Fuzhou 350108, China; 2 Institute of Precision Instrument and Intelligent Measurement & Control, Fuzhou University, Fuzhou 350108, China; 3 School of Civil Aviation, Xihang University, Xi’an 710077, China; 4 Department of Astronautic Science and Mechanics, Harbin Institute of Technology, Harbin 150001, China; 5 Hunan Toyi Carbon Material Technology Co., Ltd., Changsha 410205, China; 6 School of Automation and Intelligent Manufacturing, Southern University of Science and Technology, Shenzhen 518055, China

**Keywords:** carbon fiber reinforced plastic, top-down design approach, topology optimization, battery-pack enclosure, finite element modeling, quasi-static loading, bolt loading analysis

## Abstract

To meet the increasing demands for structural lightweighting in electric vehicles (EVs), carbon fiber reinforced plastic (CFRP) has been gradually introduced to reduce weight and enhance passenger safety in automotive engineering. The battery-pack enclosure is a key structural component for EVs, as it significantly influences the driving distance, safety, and road handling of EVs. This study presents a top-down design approach and topology optimization for a lightweight CFRP battery pack enclosure reinforced with cross-shaped stiffeners. The main objective is to develop an efficient composite enclosure that meets performance targets while accommodating the demands of cost-effective mass production. The composite battery pack enclosure was fabricated using the compression molding process. Topology optimization was carried out in the preliminary design stage on the structural shape and geometric parameters following a top-down design approach. Experimental tests recorded maximum deformations of 0.56 mm and 10.33 mm under in-plane and lateral loads, respectively. The final prototype product achieved a total mass of 4.78 kg with a rapid curing cycle of 10–15 min. In conclusion, a lightweight composite battery-pack enclosure with cross-shaped stiffeners was successfully manufactured, integrating a top-down design approach with topology optimization. This study demonstrates an effective design approach to achieving an optimal balance of lightweight, cost-effectiveness, and production efficiency for EV battery-pack enclosures.

## 1. Introduction

The demand for electric vehicles (EVs) has surged in recent years, driven by global efforts to reduce greenhouse gas emissions and transition towards sustainable transportation [1]. However, one of the persistent challenges faced by the EV industry is the limited driving range, which is significantly influenced by the vehicle’s weight [2]. The EV’s battery system, being one of the heaviest components in various serial EVs, plays a crucial role in determining the vehicle’s overall mass [3]. The battery enclosure, in particular, accounts for a substantial portion of the weight [4]. A traditional heavy-duty battery enclosure made of metals like steel or aluminum not only adds unnecessary bulk but also requires more energy to propel the vehicle [5], which leads to decreased energy efficiency and a shorter driving range [6]. As consumers increasingly demand longer-range EVs, it is necessary to develop lightweight composite battery-pack enclosures that can enhance the vehicle’s performance while maintaining safety and durability [7,8].

Carbon fiber-reinforced plastics (CFRP) have emerged as a promising material for constructing EV battery enclosures due to their unique combination of properties [9,10]. CFRP composite consists of strong carbon fiber reinforcements embedded in a polymer matrix [11]. The high strength-to-weight ratio of carbon fiber can offer tensile strengths several times higher than metallic materials [12,13]. CFRP composite allows for the creation of CFRP battery enclosures that can withstand various mechanical performances under driving operation [14,15]. Moreover, CFRP composite has excellent corrosion resistance, and it is crucial for protecting the battery system from various environmental conditions [16]. In addition, the electrical insulation and thermal properties of CFRP composite prevent electrical short circuits and provide thermal protection, which ensures the safety of the battery and the electric vehicle [17]. For example, Budiman et al. [18] investigated the phase-change composite filled in the composite enclosure for thermal protection. It was shown that the phase-change material was used as passive protection against potential thermal abuses in EV battery modules. Furthermore, composite battery enclosures can be designed to meet highly customized requirements for various serial vehicles, which effectively distributes loadings and enhances the overall structural integrity [19,20]. For instance, Ma, Q. et al. [21] introduced the parallel optimization of structural design and manufacturing feasibility of the electric vehicle battery pack. The total mass of the final pack was 14.09 kg, with a weight reduction rate of 56%. Shui et al. [22] optimized the features of the battery pack enclosure comprising four phases to determine the parameters. It is shown that the simulated design achieved a lower value of maximum deformation by 22.21%, a higher value of minimum natural frequency by 3.18%, and a decrease in mass by 11.61% from its standard design. Notably, Chen et al. [23] presented the lightweight design of CFRP and aluminum alloy with optimal target weight. It achieved the weight loss of 48.86%, and the first-order natural frequency increased to 32.03 Hz. CFRP composite is a promising material for EV composite battery-pack enclosures, which enhances structural safety with sufficient weight reduction [24].

A variety of manufacturing processes are commonly used to fabricate the lightweight composite battery enclosures, which can be broadly categorized into closed-mold and open-mold processes. Resin transfer molding (RTM) is widely used in composite battery packs. In the RTM process, dry carbon fibers are placed in a closed mold, and then a liquid resin is injected under pressure to impregnate the fibers [25]. It allows for precise control of the fiber-resin ratio and ensures a uniform distribution of the resin, resulting in a high-quality composite battery pack. In the autoclave curing process, where the molded CFRP part is placed in an autoclave, the controlled heat temperature cures the resin, while the pressure helps to eliminate voids and ensure proper consolidation of the composite [26]. For example, Bere et al. [27] manufactured the CFRP front hood concepts through an autoclave curing process for a lightweight vehicle. It was shown that the CFRP composite hood was 22% lighter than the steel hood and provided a mass reduction of about 53%. To achieve the high-performance CFRP battery enclosure with lightweight and low-cost characteristics, optimization design of lightweight battery enclosures mainly used the finite element analysis tool [28,29,30]. For instance, Lian et al. [30] focused on optimizing battery pack systems for crashworthiness and lightweight design in electric vehicles, which used the kriging surrogate model and multi-objective particle swarm algorithm. It was shown that the approach reduced the computation time by 56.2% with a practical solution for battery pack design. Liu et al. [31] constructed a digital-twin-driven lightweight design framework to achieve the aluminum enclosure as a CFRP hybrid structure. The results show that the optimized hybrid-material enclosure achieves a 12.3% weight reduction compared to the aluminum alloy structure.

As the demand for lightweight CFRP EV battery enclosures in the automotive sector grows rapidly, various recent investigations have been conducted on lightweight battery enclosures [32,33]. For example, Kulkarni et al. [9] investigated the crashworthiness of a carbon fiber ply-based EV battery enclosure, and finite element modeling was performed for the thermoforming process of the EV battery enclosure. It was highlighted that the total weight of the CFRP EV battery was 35.7% lighter than that of the Al7075-T4 aluminum alloy. Arslan et al. [34] presented the optimization and design of a glass fiber reinforced plastic (GFRP) EV battery box for lightweight and crashworthiness with a novel hybrid structure. The proposed design resulted in a structure that was 23.9% lighter, 38.6% cheaper, and 3% higher than the traditional EV battery enclosure. Furthermore, Pan et al. [35] conducted the computer-aided simulations to evaluate the crashworthiness of the battery pack enclosure. It was shown that the weight of the battery-pack enclosure was reduced, while the crashworthiness was improved as well. To better understand the potential of composite EV battery enclosures, a comparative analysis with traditional and composite materials is shown in Table 1. It is highlighted that CFRP is the premier material for EV battery enclosures when prioritizing mass reduction. It offers an exceptional strength-to-weight ratio and enables ~60% weight savings compared to metal. While the raw material cost of CFRP is high, with high manufacturing expenses, it provides a high-cost solution with a viable strategic approach to economical production. Although some works have studied optimization design for crashworthiness and lightweight purposes, there are limited works on optimization design of EV battery structures incorporating cross-shaped stiffeners.

This study presents the lightweight composite battery-pack enclosure specifically reinforced with cross-shaped stiffeners. A top-down design approach, integrating topology optimization, is employed to investigate the optimal geometric parameters and spatial distribution of these stiffeners. The composite battery enclosure was subsequently fabricated using a compression molding technique, which was selected for its suitability for high-volume, low-cost automotive production. This work aims to bridge the identified gap by developing and validating an efficient battery-pack enclosure design that leverages the specific advantages of cross-shaped stiffeners.

## 2. Materials and Methods

### 2.1. Top-Down Design Approach

**Figure 1:**
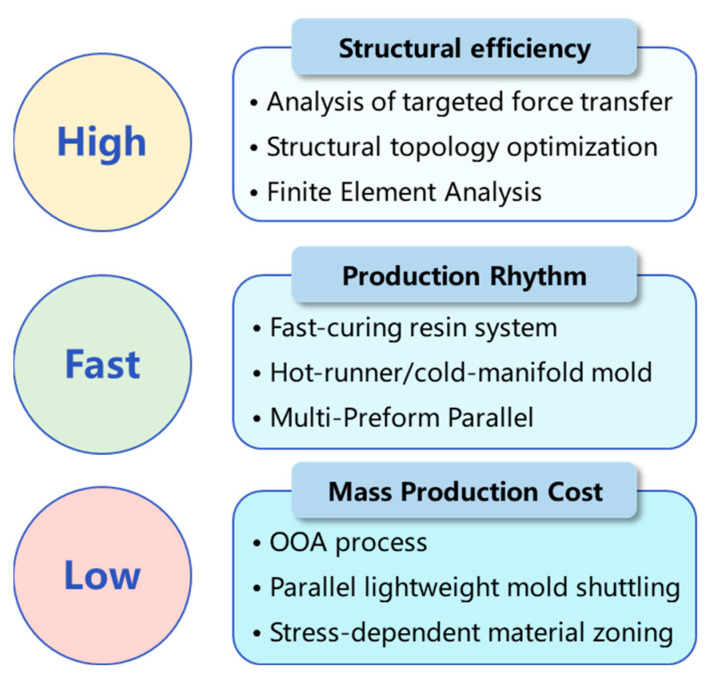
The proposed application-oriented design concept of structural efficiency, production rhythm, and mass production cost.

Figure 1 illustrates that the proposed application-oriented design concept encompasses enhancing structural efficiency, expediting production rhythm, and diminishing mass production cost. For high structural efficiency, the analysis should commence with targeted force transfer, followed by structural topology optimization and finite element modeling to assess structural integrity and rigidity. For low production rhythm, the fast-curing resin system method is utilized, integrated with hot-runner/cold-manifold molds and the multi-preform parallel approach to establish an integrated strategy. For low mass production cost, the out-of-autoclave (OOA) process is adopted, while parallel lightweight mold shuttling and stress-dependent material zoning also play a positive role.

**Figure 2:**
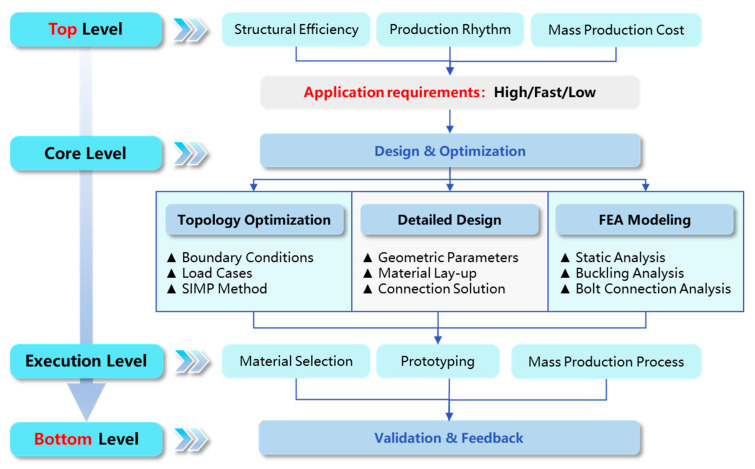
Top-down design approach of lightweight composite battery-pack enclosure.

The top-down design approach is not a single step but rather a systematic framework, that links top-level application requirements with bottom-level specific design, manufacturing, and verification, as shown in Figure 2. The top-down design approach contains the following modules: 1. Top-level: Definition and Targets; 2. Core level: Design and Optimization; 3. Execution level: Manufacturing Strategy and Execution; and 4. Down level: Experimental Validation and Closing the Loop. At the top level, from the core requirements of the new energy vehicle industry for battery pack enclosures, these are specifically categorized into three design objectives: structural efficiency, production rhythm, and mass production cost, which are the basis for all subsequent design decisions.

At the core level, topology optimization is performed. Based on defined boundary conditions and load cases, the Solid Isotropic Material with Penalization (SIMP) method is used to perform mathematical calculations within the given design space. It can determine the optimal material distribution path and identify key force-transmitting structures (e.g., cross-rib layout), which provides the core conceptual design for achieving lightweighting goals. The detailed design proceeded to translate the topology optimization results into an engineering model, which involved defined geometric parameters (e.g., size, angles, and rib layout), material layup design (e.g., CFRP/GFRP hybrid layup sequence), and connection schemes (e.g., bolt specifications and edge distances).

Finally, finite element analysis (FEA) modeling is conducted for in-depth performance verification of the proposed model, which verifies stiffness and strength, buckling analysis to assess structural stability, and bolt loading analysis to ensure connection reliability. At the implementation level, material selection begins based on cost and production efficiency objectives. Prototyping is then carried out to evaluate process feasibility. At the bottom level, the prototype undergoes bottom static load and lateral compression tests to obtain realistic load–displacement curves. Experimental results are compared with previous FEA simulation predictions to verify the accuracy of the numerical model and the reliability of the design approach. Feedback and iteration of test results are fed back into the initial design phase to refine the optimized design, which achieves a closed-loop and continuous improvement approach.

### 2.2. Topology Optimization

The SIMP method was introduced for topology optimization of a lightweight CFRP battery enclosure. A continuous design variable (density, *ρ*) was utilized to define the material distribution for the purpose of determining the primary force transmission path of the structure. The design domain is partitioned into a finite element mesh by assigning the material density of each element as the design variable (0 ≤ *ρ* ≤ 1), where *ρ* = 1 indicates the presence of solid material, *ρ* = 0 signifies a void (absence of material). If 0 < *ρ* < 1, it denotes the existence of hypothetical intermediate material that requires penalization.

The optimal layout was generated through an iterative SIMP topology optimization process, wherein the initial design domain, with the maximum allowable volume for the container, was discretized into a finite element model. Each element was assigned a pseudo-density variable that evolved to define the final geometry. Guided by a predefined objective to minimize structural compliance (i.e., maximize stiffness) under a volume fraction constraint and driven by applied boundary conditions that simulated real-world operational loads, the algorithm iteratively performed finite element analysis and sensitivity calculations. The topology optimization process systematically redistributed material penalizing intermediate densities to enforce a clear black-and-white design by removing material from low-stress regions and reinforcing high-stress load paths. The resulting “tic-tac-toe” structure on the top closure, along with the thickened base and load points, thus represented the most efficient force transmission pathways. It embodied a derived trade-off that achieved maximum stiffness with minimal material, which directly reflected the specific load cases and constraints imposed on the model.

**Figure 3:**
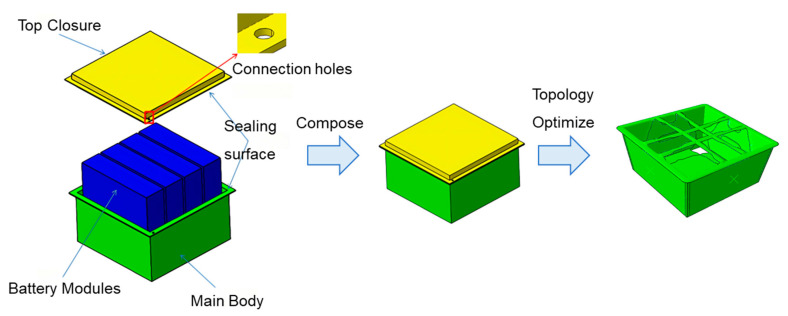
Initial composition and topology optimization of a lightweight composite battery container.

The initial model was constructed using the commercial software Abaqus/Tosca (Version 2018), which was then used to define the optimization task, design the response, define the constraints, iterate the optimization, and ultimately actualize the automatic conversion of the density field to geometry. The isotropic three-dimensional solid element model was built for the topology optimization of a lightweight FRP battery container, which was characterized by Al-6061 aluminum alloy with a 275 MPa yield strength, 68.9 GPa elastic modulus, and 2.7 g/cm^3^ density. Moreover, the 10-node modified quadratic tetrahedral solid element (C3D10M) is utilized for mathematical calculations to guarantee convergence. The result of the final topology optimization calculation is presented in Figure 3. The structural thickness is substantial toward the base and adjacent to the loading point, while the top closure is a strengthened “tic-tac-toe” structure.

### 2.3. Structural Design

**Figure 4:**
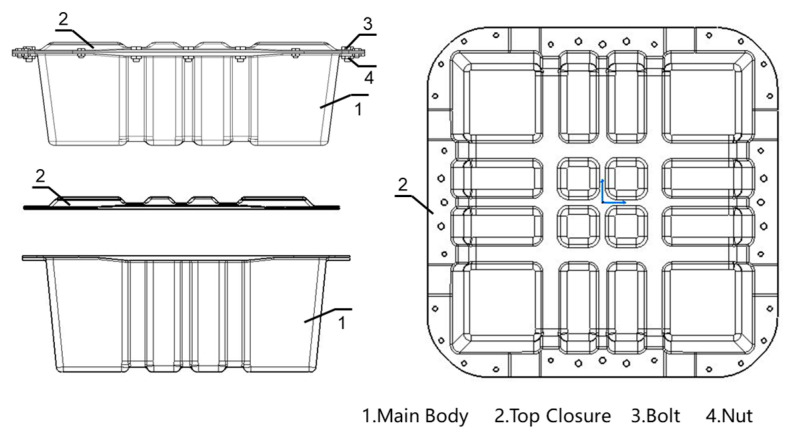
The assembly of the designed EV battery-pack enclosure with bolts and nuts.

**Figure 5:**
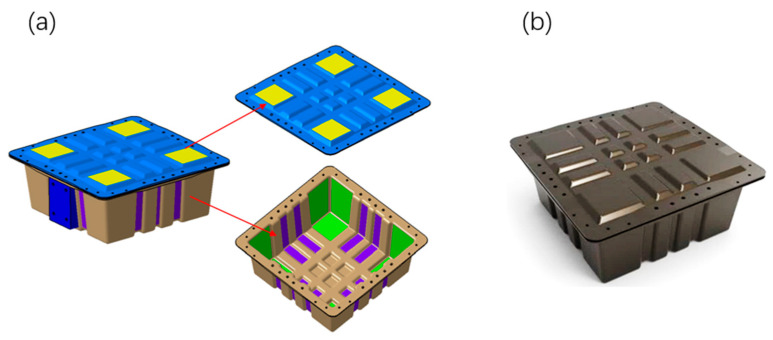
The proposed lightweight composite battery-pack enclosure: (**a**) 3D model; (**b**) manufactured product.

Based on the topology optimization results in Section 2.2 and combined with the force analysis, the structural design was carried out. The assembly of the designed battery container is shown in Figure 3. The whole container consists of four parts: the main body, the top closure, the bolts, and the nuts. Correspondingly, the 3D model is shown in Figure 4. To decrease the structural weight, the load-bearing area in the middle of the box and the lid was partially thickened (purple area) in accordance with the topological optimization result. While the thickness of the surrounding area was reduced (green area), as illustrated in Figure 5. The dimensions of the lightweight FRP battery container structure are detailed in Table 2.

To improve the structural rigidity, the battery main body and top closure are designed as an integral concave-convex reinforced structure, with the ribs arranged in a tic-tac-toe pattern. To ensure the mold design and fabrication process were in alignment, the mold was designed with concave and convex features to accomplish reinforcement. Multiple groups of Z-shaped beam structures were generated as a result of the local thickening of the reinforced area. Under the suspended static bottom loading condition, the tensile load is adequately supported by the side vertical stiffeners. Under lateral load conditions, the bending moment is borne by the side vertical reinforcement ribs, which are transmitted to the cross-shaped reinforced structure of the upper cover and the bottom. The cross-shaped reinforcement finally bears the compression loads.

### 2.4. Finite Element Modeling

#### 2.4.1. Suspended In-Plane Static Bottom Compression Modeling

**Figure 6:**
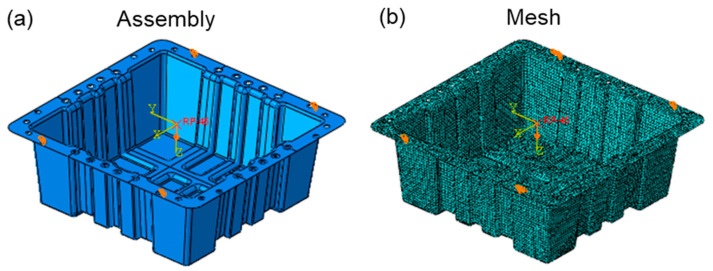
FEA verification model of the suspended static bottom loading condition: (**a**) assembly condition; (**b**) mesh condition.

The commercial Abaqus/CAE software with 2021 version was adopted for finite element analysis modeling, and the FEA model of the suspended static bottom loading condition, as shown in Figure 6. The bottom static strength verification model uses shell elements to fasten the four fixing bolt holes, which applies 1200 N on the bottom of the box. Since shell elements are used to simulate thin-walled structures, it is necessary to extract the mid-plane geometry of the bottom of the box and ignore details, such as filets, and bosses, that have little impact on the overall stiffness. The S4R four-node reduced integration shell element is suitable for general thin-walled structure analysis, which can effectively balance calculation accuracy and efficiency.

In the material property section, the CFRP material is used as an orthotropic model. Several mechanical properties of the unidirectional CFRP composite and the GFRP composite are collected in Table 3. Two surfaces are randomly selected for performing loading, the number of 0° and 90° plies in the structural layup is the same, and the number of 45° layers is the same as the number of −45° layers. Therefore, the basic carbon fiber layup sequence is [0°/45°/−45°/90°]_ns_, and a layer of glass fiber cloth is laid on the inner and outer layers. The engineering constants after the equivalent layup sequence are shown in Table 4. A load of 1200 N is applied to the outer surface of the bottom of the box in the form of uniform pressure. The pressure value is based on the bottom area of the container, which is implemented through the DLOAD command. The load direction is perpendicular to the midplane of the shell element. The static, general step is used, which enables large deformations (NLGEOM = ON) to account for geometric non-linear effects (e.g., local buckling of thin-walled structures).

#### 2.4.2. Lateral Compression Modeling

**Figure 7:**
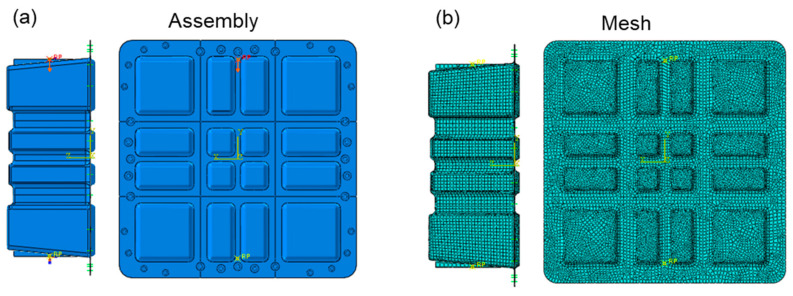
Finite element model of the lateral compression loading condition: (**a**) assembly condition; (**b**) mesh condition.

Figure 7 depicts the lateral pressure strength analysis model, which uses shell element modeling. The clamp and the box transmit the load through a contact relationship. The cover and the box are connected through a connection unit. The clamp at the lower end of the box is fixed, and a 100 kN lateral load is applied to the upper clamp. Box structure: An S4R four-node reduced integration shell element is used to simulate the CFRP thin-walled structure. Clamp modeling: The upper and lower clamps use analytical rigid body elements (Analytical Rigid), and boundary conditions and loads are applied by defining reference points (RP). For the definition of the contact pair, the contact surface between the fixture and the box adopts surface-to-surface contact. The rigid body surface of the fixture is selected as the main surface, and the outer surface of the box shell unit is selected as the secondary surface.

A Coulomb friction model is defined in the contact properties with a friction coefficient *μ* = 0.15. The automatic contact stiffness adjustment function is enabled to balance calculation accuracy and convergence. For the lower-end clamp fixation, it is applied with full constraints (U1 = U2 = U3 = UR1 = UR2 = UR3 = 0) at the lower-end clamp reference point, which binds the clamp rigid body to the box shell element through the kinematic coupling under uniform load transfer. A 100 kN lateral load is provided at the reference point of the upper end clamp, in the direction of the box width (*X*-axis). The load rises linearly to the peak value through the amplitude curve in 0.1 s to avoid convergence difficulties caused by the impact effect. The FE model is used in the static, general analysis step, which enables large deformations (NLGEOM = ON) to capture the buckling and large displacement response of thin-walled structures.

### 2.5. Fabrication Procedure

#### 2.5.1. Materials

After the structural design process was completed, the material was chosen after weighing cost and processability. To meet the demand for mass production, the selected materials need to meet the basic conditions of rapid prototyping with 5 min/150 °C. To reduce costs, a combination of glass fiber and carbon fiber prepregs was selected. The glass fiber prepreg (Y05-GF400) is a flame-retardant, low-smoke type with an areal density of 620 g/m^2^ and a plain weave pattern; it contains 37% epoxy resin. The carbon fiber prepreg (Y05-CF150) is also a flame-retardant, low-smoke type with an areal density of 230 g/m^2^ and a unidirectional tape pattern; it contains 37% epoxy resin. The selected prepregs are produced by Shenzhen No. 1 Advanced Materials Co., Ltd., Shenzhen, China, which can be quickly cured in 5 min at 150 °C.

#### 2.5.2. Fabrication of Prototype Product

Regarding the process plan and design process, production efficiency, conducting prototype trial production and cost analysis, feasibility, and economy are mainly considered. Compared with the higher production efficiency of sheet molding compound (SMC)/bulk molding compound (BMC) and efficient but high-cost high-pressure resin transfer molding (HP-RTM) technology, while excluding automated laying technologies, such as automated fiber placement (AFP)/automated tape laying (ATL), general and common molding and autoclave molding technologies are suitable in line with the design requirements. Meanwhile, it is considered that the autoclave process requires a long heating and cooling time, although multiple bodies in one cavity can improve the efficiency of a single cavity. It is considered the overall cycle of several hours or even more than 10 h, and the common cost of multiple molds, which is further compounded by the low heat conduction efficiency in the tank. A comprehensive molding strategy was adopted, which combined primary molding with negative pressure forming. This approach utilized a single mold to create multiple lightweight structures.

**Figure 8:**
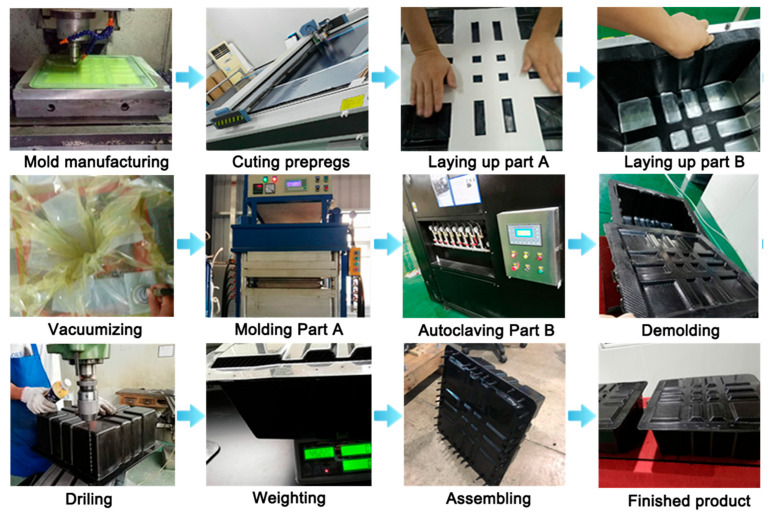
Fabrication process of lightweight CFRP battery enclosure.

Figure 8 illustrates the fabrication process of the lightweight CFRP battery enclosure. The process initiates with mold manufacturing, where a precision mold is crafted and serves as the foundational tool for shaping the CFRP components. Subsequently, cutting prepregs is performed, involving the accurate cutting of FRP prepreg materials to predefined dimensions, ensuring compatibility with the mold and design requirements. Next, the layup process for part A (top closure) and part B (main body) stages is executed. In these steps, the cut prepregs are carefully laid into the mold in a specific sequence and orientation. The layup process is critical as it determines the mechanical properties of the manufactured FRP product because it accounts for the anisotropic nature of FRP materials.

After the layup process, vacuuming is carried out to remove air from the prepreg-mold assembly, which helps in enhancing the compactness and reducing porosity in the subsequent curing stages. For the curing process, molding part A (top closure) and autoclaving part B (main body) are distinct operations. Molding part A likely involves applying heat and pressure in a mold-specific setup, while autoclaving part B is used in an autoclave environment with controlled temperature, pressure, and vacuum to cure the prepregs into rigid components. Once cured, demolding is performed to extract the hardened FRP parts from the mold without causing damage. The post-demolding and drilling process is conducted to create the necessary holes for assembly. Weighing is followed, where the components are weighed to ensure they meet the design-specified weight requirements, which is a key factor in the lightweight design of the battery container. The total weight of the battery box of this solution is 4.78 kg. Then, assembling brings the individual parts together to form the complete battery structure. Finally, the manufactured product represents the result of the entire manufacturing process. A lightweight CFRP battery-pack enclosure prototype is ready for experimental tests.

#### 2.5.3. Mass Production Process

The sample mold adopts traditional mold design and molding methods, which have low production efficiency and cannot be produced in large quantities. The sample box is formed using a single-sided mold autoclave, and only one set is produced per day, which does not meet the mass production requirements. To reduce costs and improve efficiency, mass production molds are used. Prepreg compression molding (PCM) with two inlets and two outlets for oil heating is conducted. The internal circulation design of the oil circuit enables fast heating. The ejection pin design eliminates the need for manual demolding and reduces manual demolding deformation and damage. The molding cycle (thermal curing cycle) can reach 10 min/sample, which meets certain mass production requirements. The mass production plan is used to perform multiple sets. When four sets of preform molds are designed, they are closely coordinated with the mass production molding process. It can avoid a large amount of molding time caused by the original intermittent operation process, and it improves unit time productivity and production efficiency. Optimizing mass production mold design can achieve rapid curing in 10–15 min. Meanwhile, through cost control in each link, the mass production cost of a single set can be controlled within ¥1000.

### 2.6. Experimental Test

#### 2.6.1. Suspended In-Plane Static Bottom Compression Test

**Figure 9:**
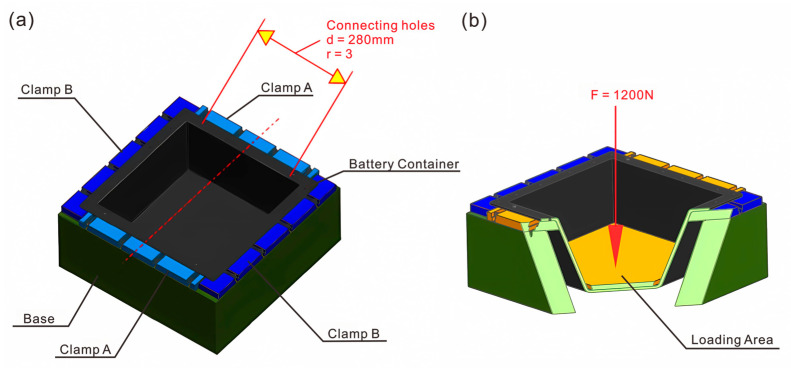
Schematic diagram of static bottom loading experimental setup: (**a**) boundary condition; (**b**) load position.

The LE5000 Universal Electronic Testing Machine from Lishi Shanghai Scientific Instrument Co., Ltd, Shanghai, China. was adopted to perform the test. As illustrated in Figure 9, the battery container assembly was affixed to the clamps, and the maximal deformation of the bottom surface of the battery container was evaluated by applying a load to the bottom. The suspended static bottom load verification was conducted in accordance with the actual operating conditions of the battery container. The container was installed in a manner that ensures that the pressure was evenly distributed based on the total weight of the battery. The experimental test procedure employed a quasi-static loading mode at a consistent rate of 2 mm/min up to 1200 N, with deformation being recorded. A battery container that was considered qualified should typically exhibit a maximum deformation of no more than 2 mm at the bottom and no visible damage to the connection holes after the bolts were removed.

#### 2.6.2. Static Lateral Compression Test

**Figure 10:**
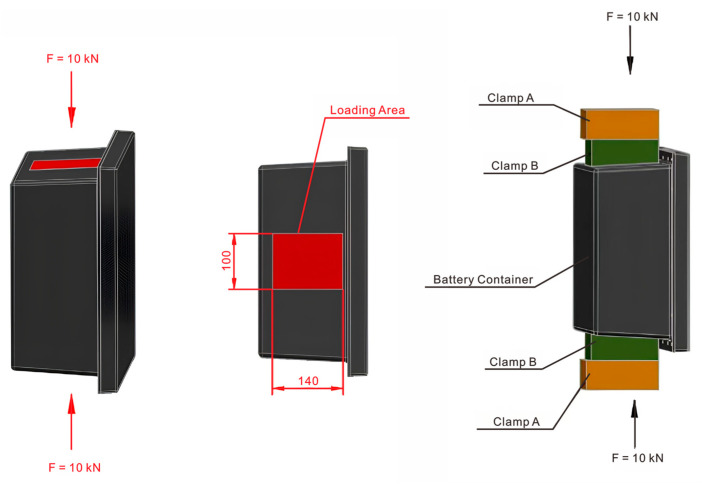
Schematic diagram of quasi-static lateral compression loading and boundary conditions.

As shown in Figure 10, the battery cap was connected and installed with the main body, and bolts were installed in all connection holes during lateral crush verification. The envelope dimensions of the displacement deformation are 330 mm × 330 mm to prevent contact with the battery. Before testing, the load was initially applied at a rate of 5 mm/min to a force of 50 N and maintained for 5 s, and the deformation was reset. The quasi-static loading method was employed, in which the load was maintained for 3 s and then unloaded. A qualified battery container should have a maximum deformation of no more than 20 mm at the base and display no apparent damage to the connecting holes following the removal of the bolts. The 10 kN lateral compression load was chosen as the standardized and targeted performance threshold to validate a critical safety requirement. Specifically, the maximum deformation under the applied load did not exceed 20 mm, and there was no visible damage after the experimental test, which provided structural integrity and occupant safety.

## 3. Results and Discussion

### 3.1. Results of Suspended Static Bottom Loading Condition

**Figure 11:**
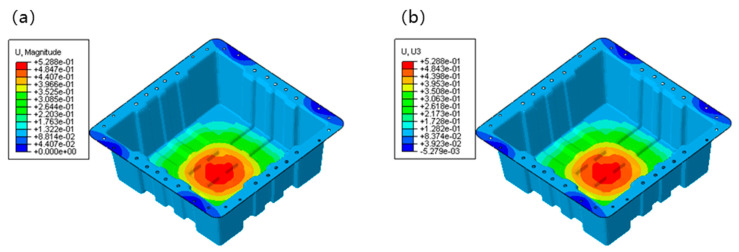
FEA results of displacement in suspended static bottom loading condition: (**a**) distribution of total displacement magnitude; (**b**) distribution of vertical displacement magnitude.

Figure 11a presents the distribution of total displacement magnitude (U, Magnitude) of the lightweight CFRP battery enclosure. A transparent gradient of displacement was observed, with the maximum displacement occurring at the central region of the container’s bottom, indicated by the red color. The maximum deformation after loading was about 0.53 mm, which met the deformation requirements of the bottom static test. This central concentration of displacement is likely due to the structural geometry and load-bearing characteristics, where the central area may experience more significant deformation under the applied loads. The displacement gradually decreased from the center to the edges, as shown by the transition from red to blue colors. The corner regions exhibited relatively low displacement, which can be attributed to the boundary constraints and the stiffening effect of the container’s peripheral structure. This deformation pattern and the achieved low displacement value are consistent with the performance of high-quality CFRP structures reported in the literature [21,23,27]. It was confirmed that similar structural efficiency was achieved with the fast-curing prepreg system, which was crucial for mass production.

Figure 11b illustrates the vertical displacement component (U, U3). Similarly to the total displacement magnitude, the maximum vertical displacement was located at the central bottom region. However, the vertical displacement distribution provided more specific information about the out-of-plane deformation. The negative values at the bottom of the color scale represented minimal downward displacements or even slight upward deformations in some local areas, which might be related to the structural stiffness and the interaction between different parts of the container. The overall trend of increasing vertical displacement from the edges to the center is consistent with the total displacement magnitude, which validates the dominance of the central region in experiencing deformation. The deformation behavior was performed through the designed FEA model, which incorporated the equivalent properties of the CFRP/GFRP hybrid laminates. It underscored the reliability of the composite modeling approach, which was aligned with similar previous findings [21]. It was emphasized that the importance of accurate material representation in FEA was for the parallel optimization of CFRP battery pack design and manufacturing.

### 3.2. Results of Lateral Loading Condition

**Figure 12:**
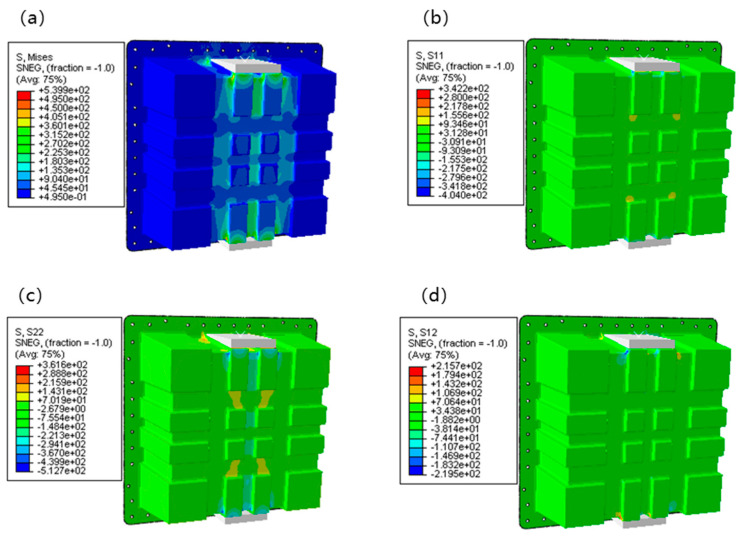
FEA results of stress in lateral loading condition: (**a**) Mises; (**b**) S_11_; (**c**) S_22_; (**d**) S_12_.

Figure 12 presents the FEA results of stress distribution for the lightweight CFRP battery container under lateral loading conditions. Four key stress components were visualized: von Mises stress (S, Mises), normal stress in the X-direction (S_11_), normal stress in the Y-direction (S_22_), and shear stress in the XY-plane (S_12_). These stress components collectively characterized the mechanical response of the CFRP battery enclosure, which was crucial for validating the top-down design approach integrating numerical modeling and topology optimization. Figure 12a depicts the equivalent stress distribution of Von Mises stress (S, Mises), which ranges from 0.495 to 5.399 × 10^2^ MPa (75% average). High-stress regions were concentrated in the central and upper-middle areas of the container, particularly around the load-bearing structure. In contrast, the peripheral and lower regions exhibited relatively low stress. This non-uniform distribution reflects the structural stiffness variation and load-transfer mechanism: the central load-bearing zone efficiently transmits and redistributes the applied load, while the outer regions mainly provide structural support with less direct load participation. Such distribution aligns with the topology optimization goal of material efficiency, as high-stress areas correspond to regions where material is strategically retained or reinforced. The efficient material distribution was a direct outcome of the topology optimization process, and with the more uniform stress distributions often reported in non-optimized metallic enclosures [4,5]. The stress levels observed within the capabilities of the selected CFRP prepreg were confirmed by the mechanical properties in Table 3. For example, Arslan et al. [34] achieved a 23.9% weight reduction with a GFRP hybrid structure, but the use of CFRP in our cross-ribbed design allows for handling higher specific loads, as evidenced by the successful withstanding of 100 kN lateral loading.

Figure 12b shows the normal stress in the X-direction (S_11_), which ranges from −0.040 to 3.422 × 10^2^ MPa (75% average). Tensile stress was observed in the middle to upper central parts, while compressive stress dominated the outer and inner regions. The alternating tensile-compressive pattern in the X-direction was attributed to the structural deformation under vertical loading, causing lateral stretching and squeezing. This stress state is critical for the CFRP structure, as the strength of the material is anisotropic. It helps verify if the fiber orientation (optimized design) aligns with the principal stress direction to maximize load-bearing capacity. The management of these normal stresses through strategic fiber layup ([0°/45°/−45°/90°]_ns_) demonstrated an advancement over some previous studies that focused primarily on geometric optimization. Kulkarni et al. [9] investigated CFRP battery enclosures but focused on ply-based thermoforming. The integrated approach of topology optimization and tailored laminate sequencing provided a more comprehensive route to structural efficiency. Figure 12c illustrates normal stress in the Y-direction (S_22_), and it shows a mix of tensile and compressive stresses, spanning from −5.127 × 10^2^ to 3.816 × 10^2^ MPa (75% average). The magnitude and pattern indicated that the vertical load-induced bending and shear effects significantly influenced the Y-direction stress. The topology optimization process likely adjusted the structural geometry to mitigate excessive stress concentrations in this direction, ensuring the CFRP container avoided premature failure (e.g., fiber-matrix debonding or fracture) under service loadings.

Figure 12d shows the shear stress in the XY-plane (S_12_) and covers −2.195 × 10^2^ to 2.157 × 10^2^ MPa (75% average). Shear stresses were distributed across the structure, with moderate magnitudes in the central load-transfer paths and lower values in the periphery. Shear stress is a key factor for CFRP structures, as interlaminar shear failure is a standard failure mode. The observed distribution validated that the topology-optimized design effectively manages shear stress transfer, leveraging the CFRP’s in-plane shear strength and avoiding critical interlaminar stress concentrations. The shear stress values were well below the shear strength of the material (Table 2), indicating a robust design. It is a critical improvement over some standard designs. For instance, Shui et al. [22] reported achieving an 11.61% mass reduction through multi-phase optimization. However, the top-down approach, starting with topology optimization, inherently minimizes shear stress concentrations from the conceptual design stage, which led to a more fundamentally sound structure. In addition, the maximum deformation of the designed structural scheme after loading was about 2.6 mm, which was far less than the deformation requirement of the lateral extrusion test. The high safety margin against instability is a significant benefit of the cross-rib stiffening pattern. It surpasses the stability often achieved in plain sheet metal enclosures or even some simpler composite designs, highlighting a key structural advantage of the proposed topology-driven approach. It aligned with the crashworthiness goals discussed by Pan et al. [35], but our method provides a predictive, simulation-driven path to achieving them early in the design process.

**Figure 13:**
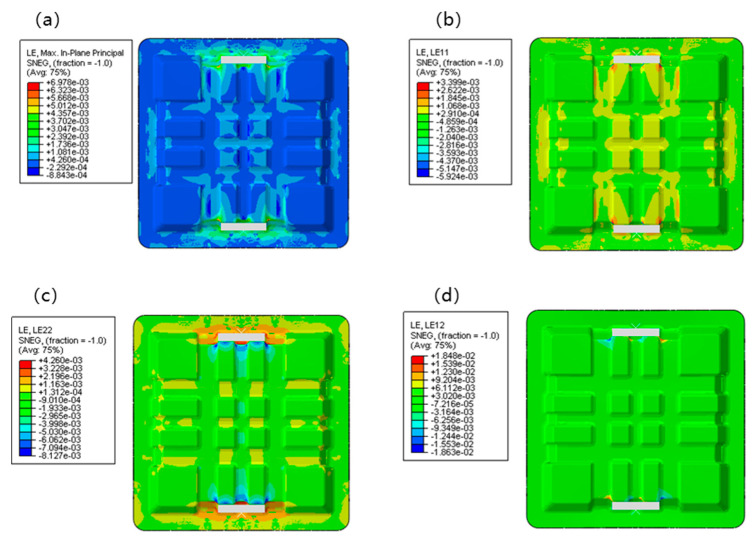
FEA results of stress in lateral loading condition: (**a**) Max. in-plane principal; (**b**) LE_11_; (**c**) LE_22_; (**d**) LE_12_.

Considering various factors, such as material shear nonlinearity, clamp deformation, the gap between the clamp and the box, and small slip, the actual test value would be slightly higher than the calculated value. The stress and strain concentration mainly occur at the bottom corners. The positive strain of the original plan is less than the material failure strain, and the shear strain is close to the failure strain. Considering that the surface of the finite element model is extracted from the inner surface of the structure, the fillet radius in the model’s corner area is smaller than that in the real structure. Therefore, the actual stress and strain levels there should be lower than the calculated values. Through finite element analysis and verification, it is determined that no structural damage has occurred, as shown in Figure 13.

**Figure 14:**
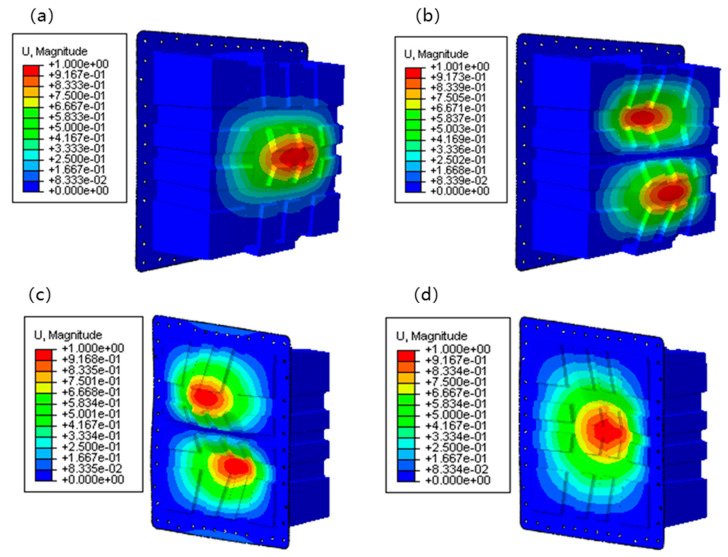
FEA results of Eigenvalue buckling loading: (**a**) first-order instability loading with 390.021 kN; (**b**) second-order instability loading with 406. 090 kN; (**c**) third-order instability loading with 509.240 kN; (**d**) fourth-order instability loading with 587.500 kN.

**Figure 15:**
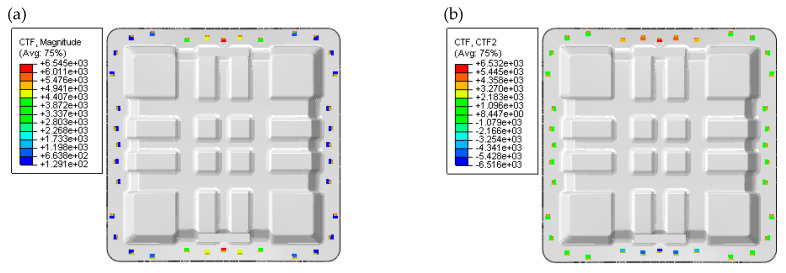
FEA results of bolt loading condition under lateral loading conditions: (**a**) total bolt load; (**b**) bolt shear load.

Due to the heavy load in the lateral loading condition, buckling instability and bolt damage were carried out to ensure the safety and integrity of the structure. Under the action of lateral load, the buckling load of the structure was much higher than 100 kN. The structure would become unstable under the loading test, as shown in Figure 14. As introduced in Figure 15, the maximum shear force on the middle bolt on the loading side was about 6500 N. The load on the middle bolt with the low-cost solution was approximately 7000 N. The bolt diameter of 8 mm was used to meet the mechanical strength requirements. The high buckling loadings calculated (first-order mode at 390 kN) significantly exceeded the operational load, which demonstrated a high safety margin. This level of stability is a marked advantage of the cross-shaped stiffener design over simpler structures and is a key benefit, as highlighted by Chen et al. [23], who reported substantial weight loss (48.86%) and improved natural frequency by combining CFRP with aluminum alloy materials.

### 3.3. Results of Experimental Verification

**Figure 16:**
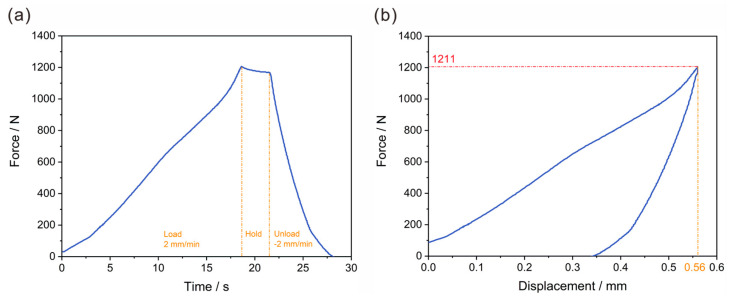
Experimental verification of the suspended static bottom loading condition: (**a**) relationship between time–force curve, and (**b**) relationship between displacement–force curve.

As Figure 16a depicts, in the initial stage (before approximately 18 s), as time progressed, the force increased continuously with a loading rate of 2 mm/min, which indicated a progressive mechanical response of the CFRP material to the applied displacement-controlled loading. Then, the force remained relatively stable around 18–21 s, suggesting that the structure reached a temporary equilibrium state under constant displacement. After that, the force decreased rapidly, which reflected the reversible and irreversible deformation characteristics in the unload stage (after 21 s) with a rate of 2 mm/min. Figure 16b presents the force-displacement relationship that indicates the force increases with displacement in a non-linear manner. The deviation from linearity also reflected the orthotropic and heterogeneous mechanical properties of the CFRP composite structure, which were considered in the numerical modeling and topology optimization process. Overall, the maximum compression force under bottom static pressure conditions was 1211.00 N, and the maximum bottom deformation was 0.56 mm. The deformation was less than the design requirement of 2 mm, and the CFRP battery pack enclosure met the requirements after the experimental test. It was implied that the CFRP structure had a specific deformation capacity before reaching a significant mechanical transition, possibly related to the onset of damage or a change in the load-bearing mechanism. The excellent agreement between the FEA-predicted deformation (0.53 mm) and the experimental result (0.56 mm) validated the accuracy of the proposed finite element model. This level of predictive capability was essential for reliable virtual prototyping and was a step forward from studies. It relies heavily on physical testing for validation, such as some previous works on composite EV battery enclosures [8].

**Figure 17:**
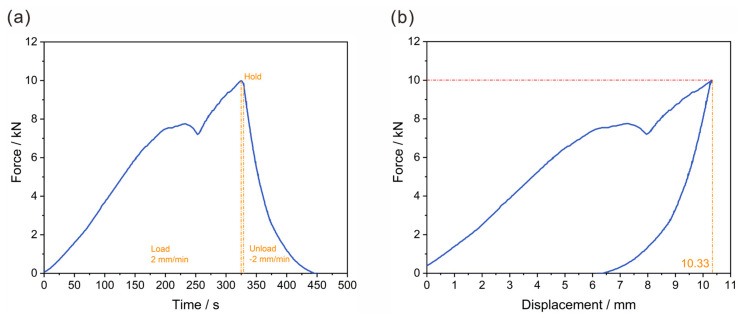
Experimental verification of the lateral loading condition: (**a**) relationship between time-force curve, and (**b**) relationship between displacement-force curve.

According to the settings in Section 2.6.2, the designed lightweight CFRP battery pack enclosure was subjected to experimental verification of the lateral loading condition. The results are shown in Figure 17, in which the two plots above illustrate the force-time and force-displacement curves. Figure 17a presents the force-time characteristics of the lightweight CFRP battery enclosure during the loading-holding-unloading cycle. In the loading phase (0–320 s), with a constant loading rate of 2 mm/min, the force increased with minor fluctuations. Around 320–340 s, the force reached a peak and remained relatively stable, implying that the structure temporarily balanced internal stresses under constant displacement. Subsequently, in the unloading phase (after 340 s) at a rate of −2 mm/min, the force declined sharply, which reflected the release of elastic and some inelastic deformations.

Figure 17b elucidates the mechanical behavior. The force grew non-linearly with displacement, and a distinct force drop was observed at a certain displacement, which corresponded to the minor fluctuation in the force-time curve. As displacement continued to increase, the force rose again until reaching a peak. Overall, the maximum compression force under lateral extrusion conditions was 100,002.00 N, and the maximum lateral deformation was 10.33 mm. The deformation was less than the design requirement of 20 mm, and the battery box met the requirements after the experimental test. Deformation performance under extreme lateral crush was noteworthy. The deformation of 10.33 mm under a 100 kN load demonstrated a high specific energy absorption, a characteristic sought after in EV battery enclosures for crashworthiness. While Pan et al. [35] focused on improving crashworthiness through design, our work proposes that a CFRP prepreg-based design with cross-shaped stiffeners can achieve comparable or superior crashworthiness targets while being inherently lightweight.

Collectively, these force-time and force-displacement curves provide comprehensive insights into the mechanical performance of the lightweight CFRP battery enclosure. The rationality of the top-down design approach is based on numerical modeling and topology optimization. It ensures that the designed structure can effectively withstand complex mechanical loads in EV operating environments. If there is contact between the clamp and the box, contact slip would occur when the friction is insufficient to resist the load. The small decrease in load in the curve around a displacement of 7.8 mm can be interpreted as a change in contact state (interface slip). No visible hole or edge damage was observed when the bolts were removed after the experimental test. The absence of damage post-test, particularly around the bolt holes, was a significant result. It is indicated that the connection design is robust, and it is a critical and challenging aspect in composite structures [31].

This study demonstrates a cost-effective strategy for manufacturing lightweight CFRP battery enclosures, contrasting with conventional metallic enclosures and high-cost composite manufacturing routes. Traditional enclosures made from steel or aluminum, while initially cheaper in raw material cost, contribute significantly to vehicle weight, thereby reducing energy efficiency and driving range [4,5]. Although advanced composite processes such as autoclave curing or HP-RTM yield high-performance parts, they involve substantial capital investment, extended cycle times, and high operational costs [17,18,19]. In contrast, the proposed approach utilizes fast-curing prepregs and compression molding with optimized multi-preform tooling, achieving a curing cycle of only 10–15 min and a target unit cost below ¥1000. It represents a significant reduction in both production time and cost compared to conventional composite methods, while still meeting structural performance targets. Similar efforts by Arslan et al. [23] highlighted cost reductions up to 38.6% using hybrid composite designs. Thus, this work not only achieves lightweight and structural efficiency but also establishes a scalable, economically viable pathway for mass production of CFRP battery enclosures.

The lightweight CFRP battery enclosure and its associated top-down design methodology present significant prospects for broader application within the EV industry and beyond. The scalability of this solution allows it to be adapted across various EV platforms, from passenger cars to light commercial vehicles, by adjusting the topology optimization parameters and geometric design to meet specific load and packaging requirements. Furthermore, the underlying principles of using stiffened, topology-optimized composite structures are not limited to battery enclosures. The proposed approach can be effectively translated to other critical vehicle components, such as structural cross-members, sub-frames, and body panels. It can contribute to a holistic vehicle lightweight strategy that enhances driving range and energy efficiency. Looking beyond electric vehicles, it holds considerable promise for the wider motor vehicle sector. The imperative for weight reduction to lower emissions and improve fuel economy in conventional internal combustion engines and hybrid vehicles makes this a universally applicable solution. The cost-effective, rapid compression molding process, which is capable of high-volume production, aligns perfectly with the automotive demands. It demonstrates a viable pathway toward next-generation, lightweight automotive structures that are both performance-driven and economically feasible.

## 4. Conclusions

This work presents a holistic engineering solution that successfully balances lightweight performance, structural integrity, and production efficiency for the lightweight battery box design. Based on the comprehensive design, finite element analysis and experimental validation are conducted, the following key conclusions are drawn:

1. An optimized lightweight structural design was successfully developed and validated. Guided by load path analysis, the process involved topology optimization followed by detailed configuration design, which led to the implementation of a cross-rib stiffened structure. The resulting design met all stringent performance criteria, including static strength, stability, and bolt connection integrity under various operational loads, and achieved a total battery box mass of 4.78 kg.
2. The prototype demonstrated excellent mechanical performance under critical loading conditions. Experimental tests confirmed the structural robustness, with a deformation of 10.33 mm under a severe 10 kN lateral crush load and a minimal deformation of 0.56 mm when subjected to a 1200 N static pressure on the base.
3. A high-efficiency and cost-effective manufacturing strategy was established for industrial mass production of the CFRP battery-pack enclosure. It was achieved by selecting fast-curing prepregs and optimizing design to achieve a rapid curing cycle of 10–15 min. Furthermore, rigorous cost-control measures across production stages ensured that the target cost per unit was below ¥1000, which guarantees its commercial market.

## Figures and Tables

**Table 1:** Comparative analysis with traditional and composite materials for EV battery enclosures.

Parameters	Steel (e.g., Mild Steel)	Aluminum (e.g., Al 6061)	GFRP	CFRP
Costs (¥/kg)	Low (~10–20)	Medium (~30–50)	Medium (~40–70)	High (~200–400)
Manufacturing cost	Low	Medium	Medium	High, but reducible
Manufacturing speed	Very high	Very high	High	Medium
Scalability for mass production	Very high	Very high	Medium	Medium to High
Thermal conductivity (W/m·K)	High (~50)	Very high (~200)	Low (~0.2–0.5)	Moderate
Mechanical properties	Low	Medium	Medium	Very high
Corrosion resistance	Low	Medium	High	High
Weight saving	Baseline	~40–50% lighter	~50% lighter	~60% or more lighter

**Table 2:** Structural dimension of the lightweight CFRP battery-pack enclosure.

Structural Parameter	Value
Length × width × height/mm	460 × 460 × 180
Angle of main enclosure body/°	5.5
Angle of slot/°	10
Angle of cover/°	10
Height × width of stiffener/mm	9 × 15
Spacing of stiffeners/mm	25
Diameter of rounding/mm	3/10
Diameter of bolt/mm	6/8
Edge distance of bolt/mm	18

**Table 3:** Mechanical properties of unidirectional CFRP and GFRP composites.

Properties	$E_{1}$ /MPa	$E_{2}$ /MPa	$\nu_{12}$	$G_{12}$ /MPa	$G_{13}$ /MPa	$G_{23}$ /MPa
CFRP	131,000	8180	0.3	5320	5320	3476
GFRP	22,800	22,800	0.142	3800	3800	3000

**Table 4:** Equivalent mechanical properties of CFRP/GCFP composite laminates.

Equivalent Properties	$E_{1}$ /MPa	$E_{2}$ /MPa	$\nu_{12}$	$G_{12}$ /MPa	$G_{13}$ /MPa	$G_{23}$ /MPa
Composite laminates	50,771	50,771	0.3	19,538	4560	4560

## Data Availability

The original contributions presented in this study are included in the article. Further inquiries can be directed to the corresponding authors.

## Abbreviations

AFP	Automated fiber placement
ATL	Automated tape laying
BMC	Bulk molding compound
CAE	Computer-aided engineering
CFRP	Carbon fiber reinforced plastic
C3D10M	Modified quadratic tetrahedral element
EVs	Electric vehicles
FEA	Finite element analysis
FRP	Fiber reinforced plastic
GFRP	Glass fiber reinforced plastic
HP-RTM	High-pressure resin transfer molding
OOA	Out-of-autoclave
PCM	Prepreg compression molding
RP	Reference point
RTM	Resin transfer molding
SIMP	Solid isotropic material with penalization
SMC	Sheet molding compound
